# Supplementary material for: Cobamide-mediated enzymatic reductive dehalogenation via long-range electron transfer
Source: Nat Commun. 2017 Jul 3;8:15858. doi: 10.1038/ncomms15858 (PMC5500849; doi:10.1038/ncomms15858)
Supplement: Supplementary Information — Supplementary Figures, Supplementary Tables and Supplementary References [file ncomms15858-s1.pdf]

SUPPLEMENTARY INFORMATION

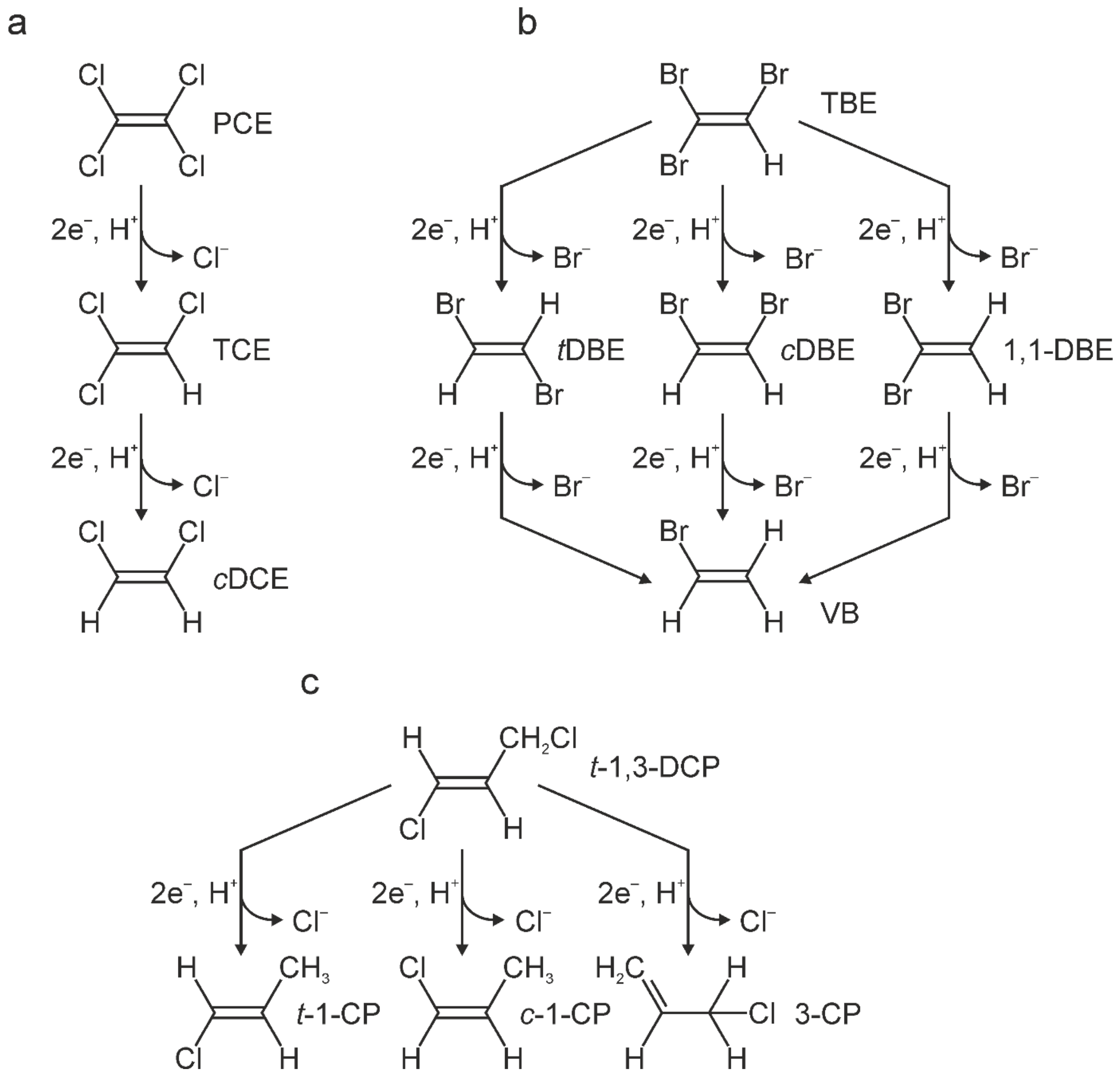

Supplementary Figure 1: Conversion of tetrachloroethene (a), tribromoethene (b), and *trans*-1,3-dichloropropene (c) by PceA. PCE: perchloroethylene / tetrachloroethene, TCE: trichloroethene, *c*DCE: *cis*-1,2-dichloroethene, TBE: tribromoethene, *t*DBE: *trans*-1,2-dibromoethene, *c*DBE: *cis*-1,2-dibromoethene, 1,1-DBE: 1,1-dibromoethene, VB: vinylbromide, *t*-1,3-DCP: *trans*-1,3-dichloropropene, *t*-1-CP: *trans*-1-chloropropene, *c*-1-CP: *cis*-1-chloropropene, 3-CP: 3-chloropropene.

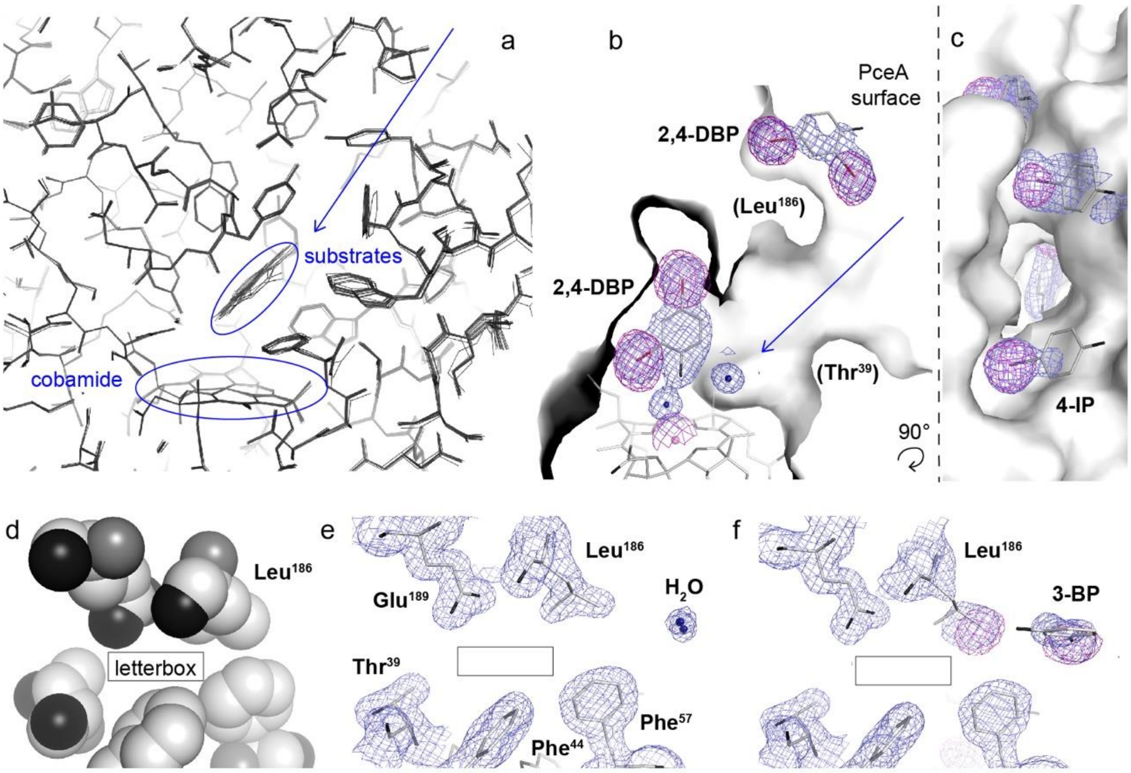

Supplementary Figure 2: Access of halogenated phenols to the substrate channel and binding pocket of PceA. (a) Slice-through PceA highlighting the substrate channel (arrow), norpseudo-B_{12} cofactor, and substrate binding site. All structures presented here (see Table 2) were overlayed. (b) Active site cavity and substrate channel with 2,4-DBP bound in both the active site and on the protein surface. Two representative water molecules (blue spheres) close to Co and the substrate are shown. Additional water and buffer molecules within the substrate channel and protein surface are omitted for clarity. The size of the substrate entrance is restricted to a maximum width of 5.5 Å by *inter alia* Leu186 and Thr39. (c) “Letter box” opening of the substrate channel of PceA with 4-IP bound to the hydrophobic groove at the protein surface. (d) Van-der-Waals radius representation of the substrate entry letter box. (e) Defined electron density for letter box residues (2,4,6-TBP bound PceA shown as representative structure). (f) Electron density for 3-BP bound PceA is perturbed at Leu186. Anomalous signal indicates partial replacement at the common Leu186 position with a 3-BP molecule.

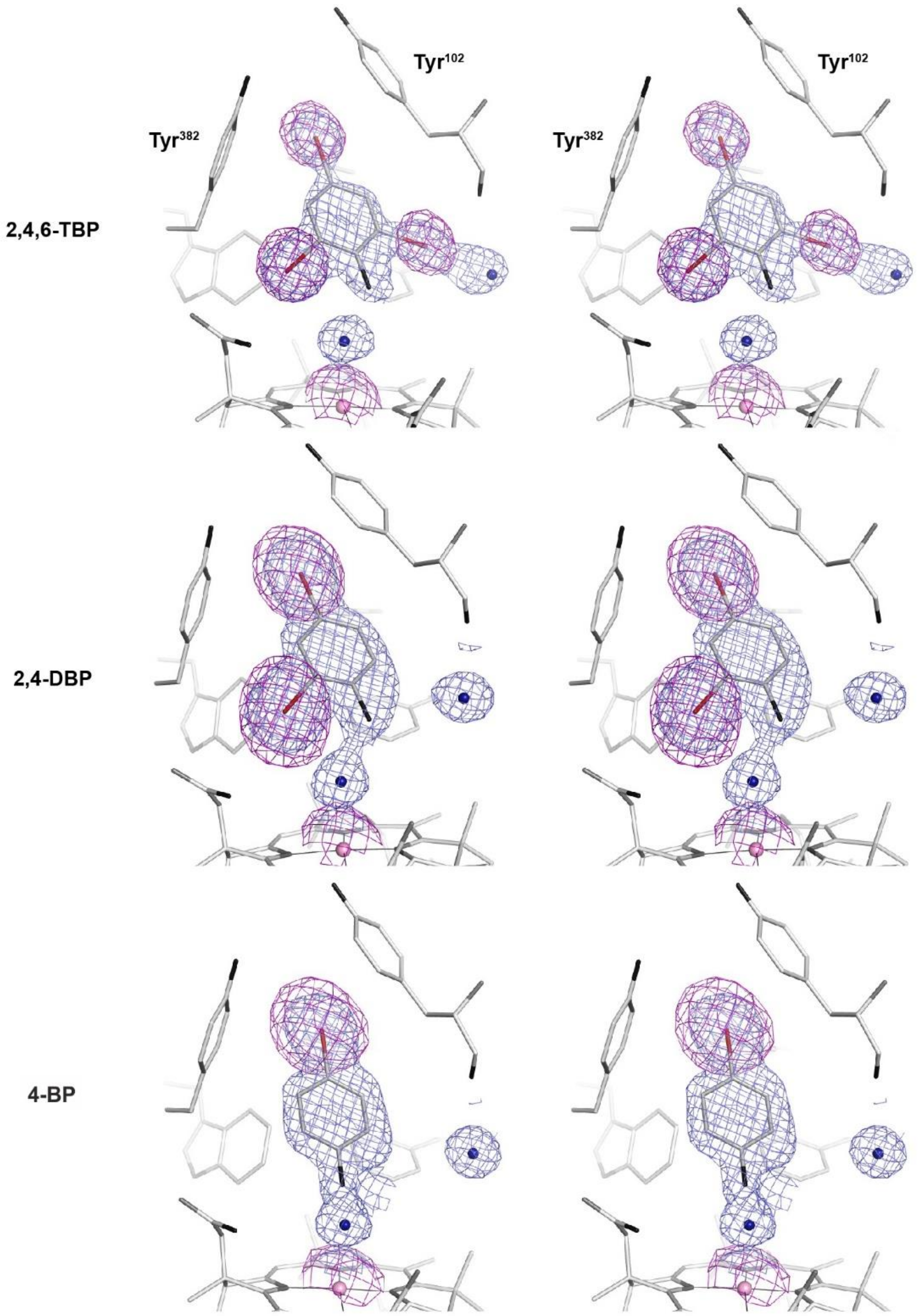

Supplementary Figure 3: Wall-eye stereo representation of 2,4,6-TBP, 2,4-DBP, and 4-BP bound to PceA. Blue mesh represents 1 σ 2F_o-F_c electron density maps and purple mesh 5 σ anomalous difference density maps at λ = 0.918 Å, indicative of bromide (red sticks). The substrate hydroxyl group is shown in black, the cobalt atom in pink.

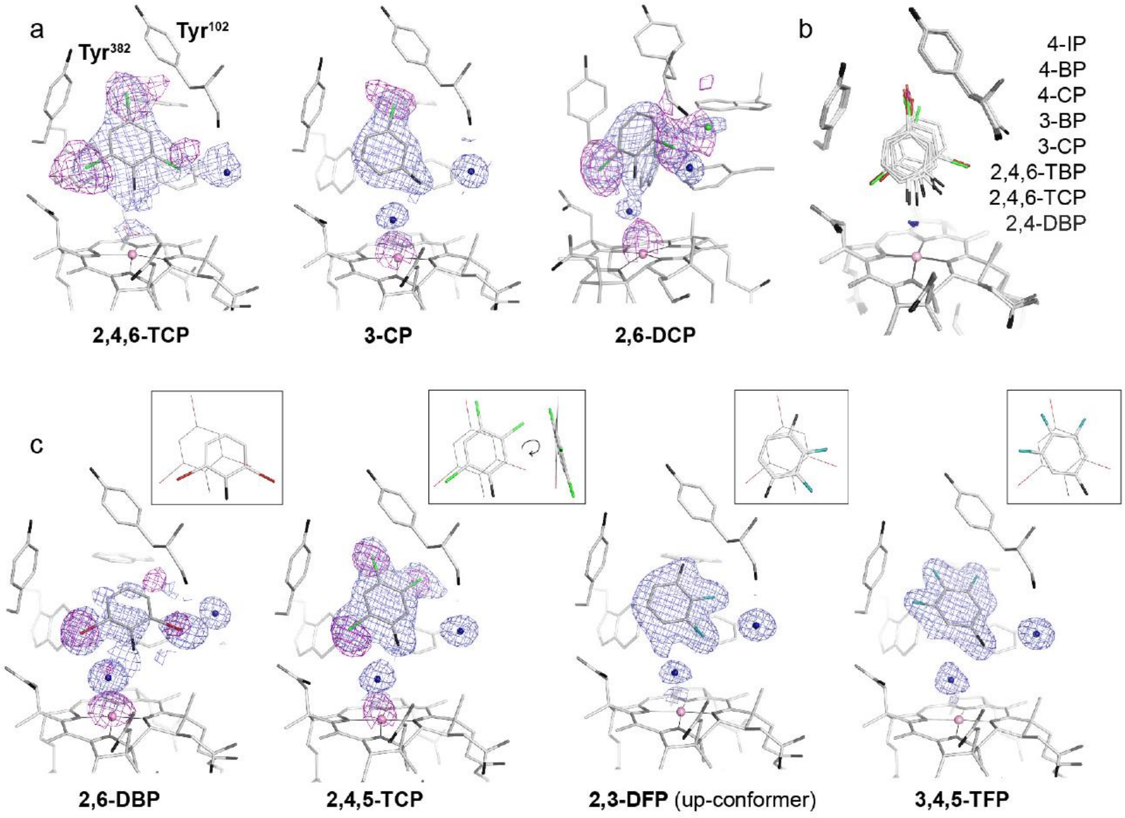

Supplementary Figure 4: Positioning of halogenated phenols in the substrate-binding pocket of PceA. (a) 2,4,6-TCP*, 3-CP*, and 2,6-DCP share the same position as their brominated analogues, but were not converted by PceA. Electron density for the chloride atom is dispersed away from C6, but not C2 in 2,6-DCP. No cobalt β-ligand was modelled for 2,4,6-TCP because of the lower resolution. (b) Overlay of tested halogenated phenols demonstrates a similar positioning in the active site. (c) Substrates bound in different orientations: 2,6-DBP* shifted towards Tyr102. 2,4,5-TCP pitched 15° backwards to circumvent the close proximity between the chlorine at C5 and Tyr102. 2,3-Difluorophenol is apparently bound in two orientations that are inverted horizontally (see inset). 3,4,5-Trifluorophenol is able to orient its *meta*-fluorines towards the inhibited position close

to Tyr382 and also towards Tyr102. The smaller fluorine substituents seem to fit into these positions.

In the direction shown, Tyr382, Tyr102, and the corrin ring encircle the binding site. For all substrates 1 sigma $2F_o$-F_c electron density (blue) for substrate, Co-ligand water, and the first water molecule in the substrate channel are shown. Anomalous difference density (indicative of a heavy atom) is shown around the substrate for bromine (5 sigma) or chlorine (3.5 sigma for 2,4,5-TCP and 3-CP, 3.0 sigma for 2,6-DCP and 2.5 sigma for 2,4,6-TCP; data were collected at 1.9 Å X-ray wavelength). Selected overlays are shown as insets. Substrates marked with * were incubated together with crystals in buffer containing 200 mM chloride as counter ion.

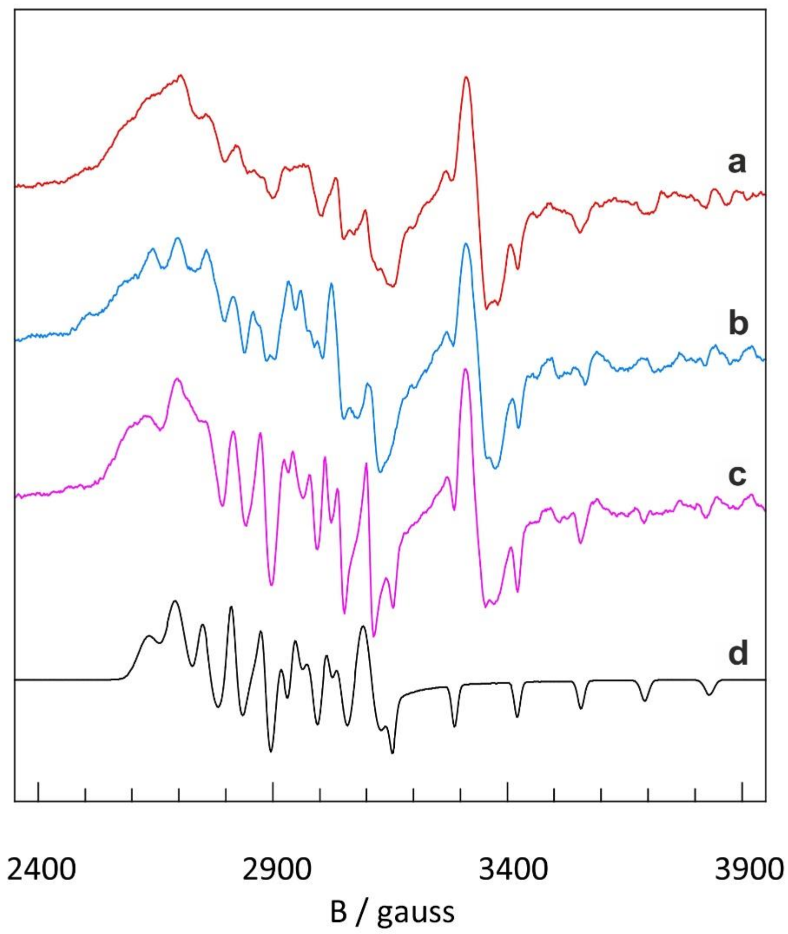

Supplementary Figure 5: Overview of spectral effects in the [Co^{II}]-EPR of PceA upon incubation with substrates. Trace a is the low-spin S = 1/2 spectrum of the cobamide cofactor in as-isolated enzyme (61 µM). The feature near g = 2 (circa 3350 gauss) is from a minor [3Fe-4S] species, a degradation product of the [4Fe-4S] clusters. Trace b shows the spectrum after 2 min incubation in a saturated PCE solution, which led to a minor increase in resolution of the I = 7/2 Co nuclear hyperfine interaction (also observed for other halogenated ethenes). A further increase in resolution in particular in the middle part of the spectrum is afforded upon incubation with the phenol derivatives; shown is 2,4-DBP (trace c). The latter spectrum is simulated in trace d with parameters (zyx-value): g-values 1.989, 2.292, 2.338; hyperfine A-values 134, 55, 65 gauss; linewidths W 10, 25, 25 gauss, line asymmetry values B 0.03, -0.04, -0.04 and C 0.03, 0.03, 0.03. The latter are defined

as total linewidth = $W(1+Bm_I+Cm_I^2)$, in which m_I is the nuclear quantum orientation running from -7/2 to 7/2 for cobalt[1]. The spectral sharpening upon substrate addition may be partly due to a general attenuating effect of the organic substrate on ice crystal growth during freezing of the samples in liquid nitrogen (*i.e.,* causing a reduction of g-strain), but the conspicuous grouping of the spectra in ethene- versus phenol-type would seem to indicate that there is also a more specific effect on active-site conformation upon substrate binding.

Experimental EPR conditions were: microwave frequency, 9338 MHz; microwave power, 12.7 mW; modulation frequency, 100 kHz, modulation amplitude, 8 gauss; temperature 22 K.

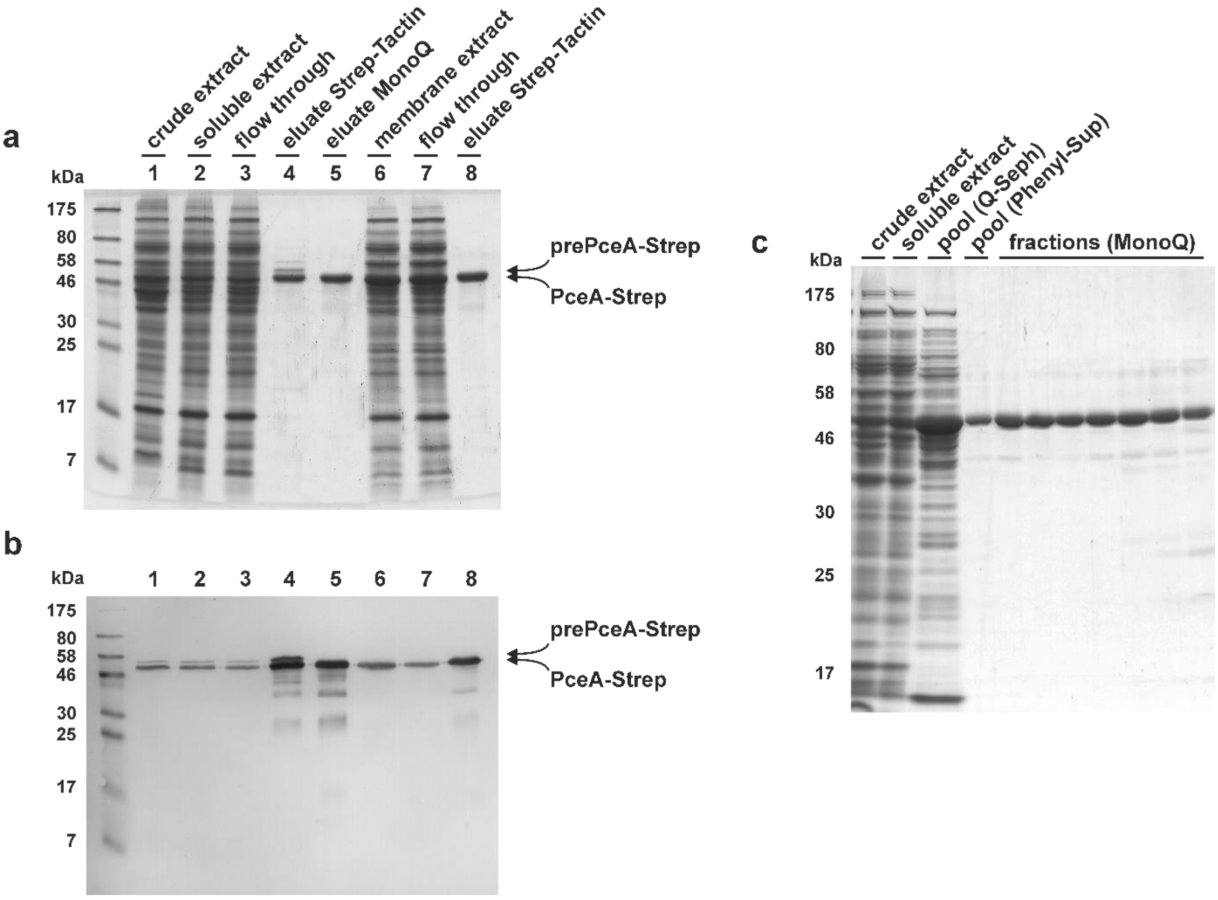

Supplementary Figure 6: Purification of recombinant PceA-Strep from *S. multivorans* GD21 and purification of PceA from the wild type strain. (a) PceA-Strep was isolated from soluble and membrane extract of *S. multivorans* GD21, grown on pyruvate/PCE medium in the presence of 720 µM $FeSO_4$, via Strep-Tactin affinity-chromatography. An apparently homogenous mature PceA-Strep protein was isolated, when the membrane extract was applied (lane 8). A mixture of the enzyme's precursor prePceA-Strep and the mature form was obtained when soluble extract was used (lane 4). The binding of the affinity-tagged enzyme to the Strep-Tactin matrix appeared to be very loose, since a significant portion of the PceA-Strep was detected in the flow through of the column. This observation has been made previously, when the C-terminally Strep-tagged PceA of *Dehalobacter restrictus* was heterologously produced and purified via affinity chromatography[2]. Both results point

towards a C-terminus of PceA enclosed by the protein structure with little access to the surrounding. Coomassie-stained 12.5% SDS-PAGE with 10 µg protein (except the eluates, of which 1 µg was applied) separated on each lane. (b) Immunoblot of the SDS-PAGE developed with an antibody against the Strep-tag. (c) PceA was conventionally isolated from the soluble extract of *S. multivorans*, grown on pyruvate/fumarate medium in the presence of 90 µM $FeSO_4$, via a combination of Q-Sepharose, Phenyl-Superose, and MonoQ column[3]. Coomassie-stained 12.5% SDS-PAGE with 10 µg protein of extract and from pooled fractions eluted from a Q-Sepharose column and 1 µg protein from pooled fractions eluted from a Phenyl-Superose column and from eluates of a MonoQ column, respectively, separated on each lane.

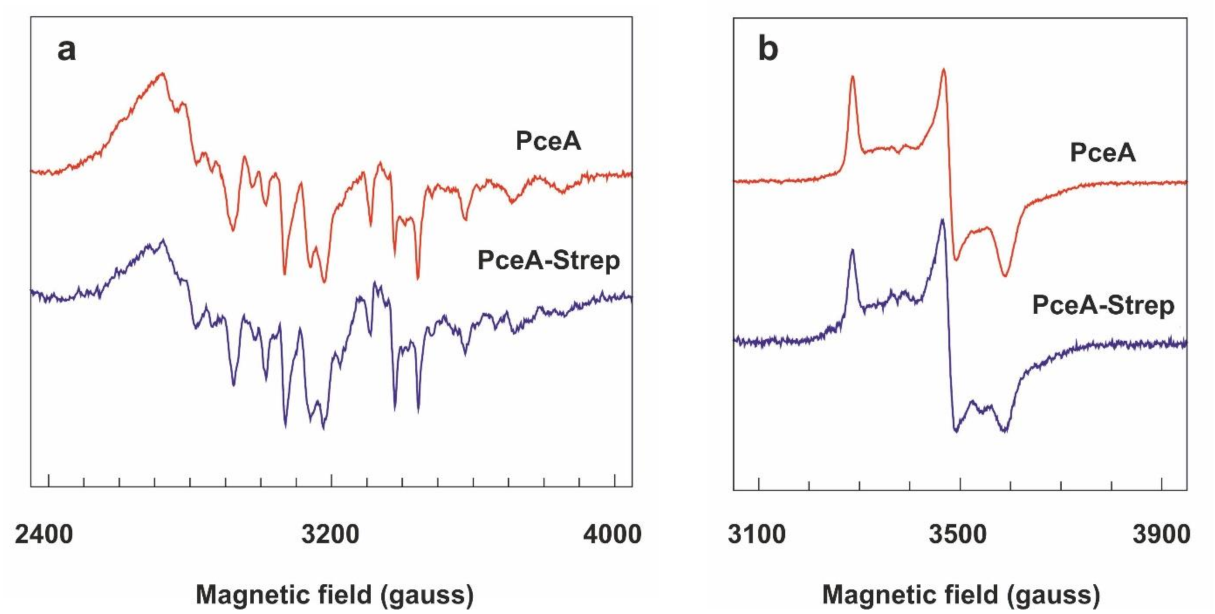

Supplementary Figure 7: Determination of the redox states of the metal-cofactors in mature PceA (red traces) and mature PceA-Strep (blue traces) by EPR spectroscopy. (a) Cob(II)alamin spectra of as-isolated enzyme. The two spectra were essentially identical and were typical for cob(II)alamin in the base-off configuration as indicated by the lack of characteristic superhyperfine splitting from an axially coordinating nitrogen base. The base-off configuration of the cobamide cofactor in the wild type PceA enzyme has been reported earlier[6]. Estimation of the spin Hamiltonian parameters by simulation[1] afforded g-values of $g_{||} = 2.00$ and $g_{\perp} \approx 2.32$ and cobalt hyperfine (I = 7/2) values of $A_{||} = 132$ and $A_{\perp} \approx 60$ gauss. These values were quite similar to those previously found for the base-off form of cob(II)alamin in the PCE reductive dehalogenase of *Dehalobacter* restrictus[7] or in the *ortho*-chlorophenol reductive dehalogenase of *Desulfitobacterium* dehalogenans[8]. Application of elevated positive redox potentials up to 309 mV by the addition of potassium hexacyanoferrate(III) did not led to the disappearance of the [Co^{II}] signal, which would indicate [Co^{III}] formation.

(b) [4Fe-4S] spectra for dithionite-reduced enzyme. Upon reduction with sodium dithionite the $[Co^{II}]$ signal disappeared, because of $[Co^{I}]$ formation. At 17 K the characteristic interaction pattern appeared for two $[4Fe\text{-}4S]^{1+}$ clusters with mutual dipolar coupling over a typical distance of about 10 Å. The effective g-values read from the spectra were g_z = 2.05, g_y = 1.94, g_x = 1.88, but these were likely to be shifted somewhat from the actual g-values of the cubanes by virtue of the dipolar interaction.

EPR conditions were: microwave frequency, 9448 MHz; microwave power, -22 dB (1.26 mW) for trace a and -26 dB (0.5 mW) for trace b; temperature, 31 K for trace a and 17 K for trace b.

Spin quantification versus an external copper(II)-standard gave concentrations of 166 μM cobalt and 355 μM cubane for the sample containing PceA and 83 μM cobalt and 155 μM cubane for the sample containing PceA-Strep, *i.e.,* two iron-sulfur clusters per cobamide cofactor in both preparations. These results were in accordance with previously reported 1 mol cobamide and 8 mol iron per mol PceA[4] or PceA-Strep (Supplementary Table 2). From the spectroscopic results obtained for the PceA-Strep variant in comparison to the wild type PceA a negative effect of the C-terminal Strep-tag on the maturation and activity of the enzyme was excluded.

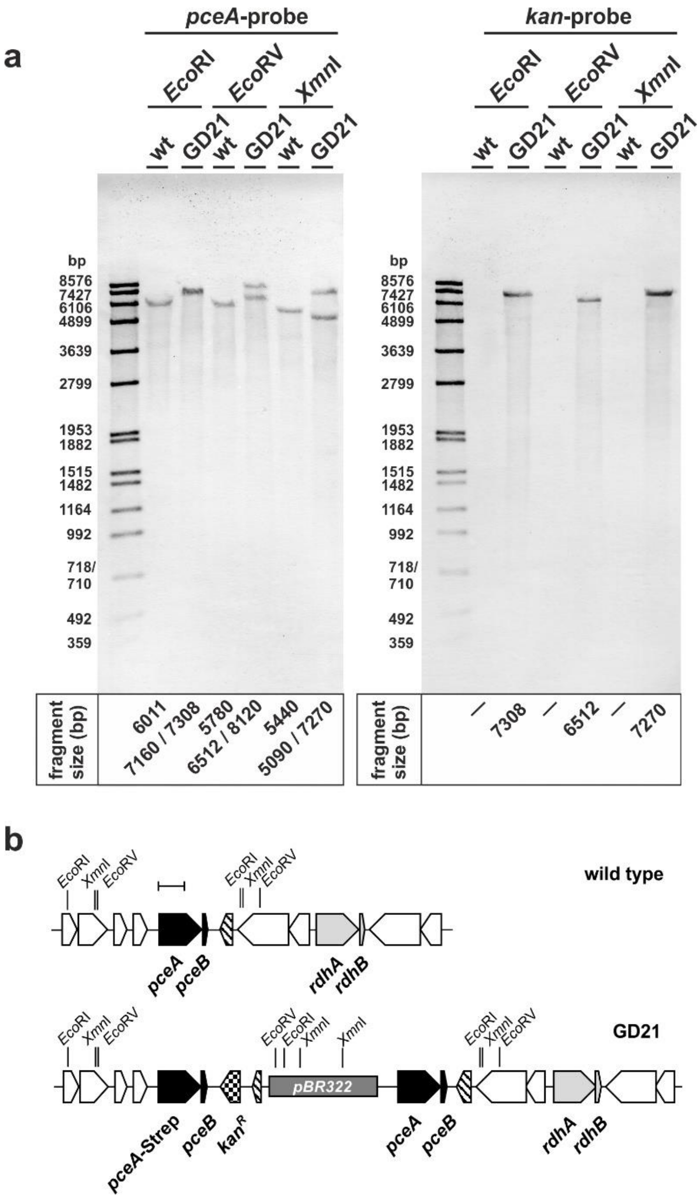

Supplementary Figure 8: Generation of the mutant strain *S. multivorans* GD21 by incorporation of a modified *pceA* gene cluster into *S. multivorans* via single homologous recombination. (a) The integration of the plasmid into the organohalide respiration region in the genome sequence of *S. multivorans* wild type (Genbank accession no. CP007201)[12] was proven by Southern hybridization analysis of *S. multivorans* GD21 in comparison to the wild type (wt) using specific DNA probes against *pceA* and *kanamycin (kan)*. No impact on PCE dependent growth was

observed for the mutant strain compared to the wild type. (b) Schematic representation of the genetic manipulation. The scale bar represents 1000 base pairs.

Supplementary Table 1: PceA activity for 4-BP in the presence of different artificial electron donors. All viologens (0.5 mM) were reduced by the addition of 1.4 mM Ti(III) citrate. Sodium dithionite was used as sole electron donor in a concentration of 1.4 mM without further addition of Ti(III) citrate. Given are relative specific activities in relation to the standardised activity with methyl viologen as electron donor (100%).

The use of sodium dithionite as reducing agent showed 14 times slower conversion rates compared to benzyl viologen as reducing agent with a similar redox potential. In PceA crystals treated with 5 mM dithionite, a heart-shaped electron density attributed to a SO_2 molecule with the sulphur atom pointing to the Co was found at the active site, which might have caused the reduced activity.

Electron donor	$E^{\circ'}$ (mV)	Relative specific activity (%)
Ethyl viologen	-480	167
Methyl viologen	-440	100
Sodium dithionite	-386	2.5
Benzyl viologen	-360	34

Supplementary Table 2: Cofactor content in isolated PceA-Strep from *S. multivorans* GD21 cultivated in the presence of different $FeSO_4$ concentrations. The conventionally purified PceA from *S. multivorans* wild type contained about 8 mol iron and 1 mol cobamide per mol purified PceA when grown in the presence of 90 µM $FeSO_4$[4]. Under these conditions, only 2.9±0.1 mol iron and 0.3 mol cobamide were detected per mol PceA-Strep. Since *S. multivorans* produces the cobamide cofactor *de novo* in high excess, the limitation might be due to a shortage in iron supply rather than in cobamide production. An increase of the $FeSO_4$ concentration up to 720 µM in the growth medium led to the production of mature PceA-Strep completely occupied with cofactors. The iron limitation may be caused by the parallel expression of two *pceA* genes in strain GD21 (Supplementary Fig. 8). The cobamide cofactor was extracted from PceA-Strep and analysed via high-performance liquid chromatography as described by Keller *et al.*[3]. Iron determination was performed using the method published by Fish[5].

[$FeSO_4$] (µM) in the medium	Metals/Cofactors (mol/mol PceA-Strep)	
	n [Fe]	n [Cobamide]
PceA Neumann *et al.*, 1996[4]	8	1
S. multivorans GD21		
90	2.9 ± 0.1	0.3
180	4.35 ± 0.25	n.d.
360	4.45 ± 0.25	n.d.
720	7.5 ± 0.4*	1.0*
	7.1 ± 1.2#	1.0#

At a $FeSO_4$ concentration of 720 µM the cofactors were separately extracted from PceA-Strep isolated from soluble extract (*) or membrane extract (#).
n.d.: not determined

Supplementary Table 3 part 1/4. Data collection and refinement statistics (molecular replacement)

	3-BP (5MAA)	3-CP (5M8Y)	4-BP (5M8U)	4-IP (5MA2)
**Data collection**				
Space group	$P\ 4_1$	$P\ 4_1$	$P\ 4_1$	$P\ 4_1$
Cell dimensions				
a, *b*, *c* (Å)	73.8, 73.8,185.4	73.8, 73.8,185.2	73.6, 73.6, 184.6	73.8, 73.8 184.7
α, β, γ (°)	90, 90, 90	90, 90, 90	90, 90, 90	90, 90, 90
Resolution (Å)	45.5 - 1.69 (1.75 - 1.69)	47.4 - 1.86 (1.92 - 1.86)	36.8 - 1.90 (1.97 - 1.90)	45.4 - 1.88 (1.95 - 1.88)
R_{merge} (%)	9.1 (122)	5.4 (37)	9.9 (95)	8.2 (39)
$I / \sigma I$	21.6 (2.0)	29.1 (3.3)	19.9 (2.3)	19.3 (2.9)
Completeness (%)	100 (99)	97 (78)	100 (100)	99 (89)
Redundancy	13.8 (13.1)	10.8 (4.4)	7.7 (7.7)	11.0 (4.8)
**Refinement**				
Resolution (Å)	45.5 - 1.69	47.4 - 1.86	36.8 - 1.90	45.4 - 1.88
No. reflections	110982	80946	76762	79047
R_{work} / R_{free}	14.8 / 16.7	13.0 / 16.1	14.6 / 17.2	13.6 / 16.5
No. atoms				
Protein	6852	6857	6866	6858
Ligand/ion	277	274	299	322
Water	759	763	783	721
B-factors (Å^2)				
Protein	28	29	28	32
Ligand/ion	26	28	29	34
Water	39	40	41	43
R.m.s. deviations				
Bond lengths (Å)	0.010	0.007	0.008	0.011
Bond angles (°)	1.86	0.92	0.91	1.88

*Values in parentheses are for highest-resolution shell.

Supplementary Table 3 part 2/4. Data collection and refinement statistics (molecular replacement)

	4-CP (5M8W)	2,4-DBP (5M92)	2,6-DBP (5M91)
**Data collection**			
Space group	$P\ 4_1$	$P\ 4_1$	$P\ 4_1$
Cell dimensions			
a, *b*, *c* (Å)	73.3, 73.3, 184.4	73.9, 73.9, 185.1	73.5, 73.5, 184.6
α, β, γ (°)	90, 90, 90	90, 90, 90	90, 90, 90
Resolution (Å)	39.0 - 2.28 (2.36 - 2.28)	46.3 - 1.79 (1.85 - 1.79)	30.1 - 1.72 (1.78 - 1.72)
R_{merge} (%)	15.0 (89)	9.0 (95)	6.7 (76)
$I / \sigma I$	19.3 (2.6)	22.1 (2.4)	22.1 (2.6)
Completeness (%)	100 (099)	100 (96)	100 (99)
Redundancy	7.4 (7.1)	13.6 (13.4)	7.7 (7.6)
**Refinement**			
Resolution (Å)	39.0 - 2.28	46.3 - 1.79	30.1 - 1.72
No. reflections	44129	93432	103500
R_{work} / R_{free}	14.0 / 19.3	14.9 / 17.8	14.6 / 17.5
No. atoms			
Protein	6837	6857	6855
Ligand/ion	301	316	279
Water	500	731	801
B-factors (Å^2)			
Protein	32	29	27
Ligand/ion	34	30	27
Water	38	41	39
R.m.s. deviations			
Bond lengths (Å)	0.008	0.029	0.008
Bond angles (°)	0.93	0.93	0.96

*Values in parentheses are for highest-resolution shell.

Supplementary Table 3 part 3/4. Data collection and refinement statistics (molecular replacement)

	2,4,6-TBP (5M2G)	2,6-DCP (5MA0)	2,4,6-TCP (5MA1)
**Data collection**			
Space group	$P\ 4_1$	$P\ 4_1$	$P\ 4_1$
Cell dimensions			
a, *b*, *c* (Å)	73.6, 73.6, 185.0	73.6, 73.6, 184.5	73.5, 73.5, 179.4
α, β, γ (°)	90, 90, 90	90, 90, 90	90, 90, 90
Resolution (Å)	47.3 - 1.80 (1.86 - 1.80)	31.0 - 1.90 (1.97 - 1.90)	33.9 - 2.6 (2.69 - 2.60)
R_{merge} (%)	7.2 (71)	6.6 (80)	17.7 (128)
$I / \sigma I$	15.1 (2.1)	32.3 (2.7)	20.0 (2.3)
Completeness (%)	100 (100)	99 (96)	100 (100)
Redundancy	4.6 (4.7)	14.1 (9.4)	15.0 (14.7)
**Refinement**			
Resolution (Å)	47.3 - 1.80	31.0 - 1.90	33.9 - 2.60
No. reflections	90629	76268	29139
R_{work} / R_{free}	13.4 / 16.7	13.6 / 16.7	15.0 / 21.5
No. atoms			
Protein	6863	6828	6559
Ligand/ion	290	264	242
Water	771	681	215
B-factors (Å^2)			
Protein	28	29	46
Ligand/ion	28	27	40
Water	42	40	45
R.m.s. deviations			
Bond lengths (Å)	0.010	0.007	0.010
Bond angles (°)	1.00	0.94	1.15

*Values in parentheses are for highest-resolution shell.

Supplementary Table 3 part 4/4. Data collection and refinement statistics (molecular replacement)

	2,4,5-TCP (5M8X)	2,3-DFP (5M8Z)	3,4,5-TFP (5M90)
**Data collection**			
Space group	$P\,4_1$	$P\,4_1$	$P\,4_1$
Cell dimensions			
a, *b*, *c* (Å)	73.8, 73.8, 185.0	73.6, 73.6, 185.0	73.6, 73.6, 185.1
α, β, γ (°)	90, 90, 90	90 90 90	90, 90, 90
Resolution (Å)	47.3 - 1.87 (1.94 - 1.87)	47.3 - 1.66 (1.72 - 1.66)	47.3 - 1.60 (1.66 - 1.60)
R_{merge} (%)	5.8 (29)	5.2 (62)	4.8 (63)
I / σI	26.7 (4.0)	21.9 (2.8)	24.9 (2.8)
Completeness (%)	97 (75)	100 (100)	1.00 (99)
Redundancy	11.2 (5.3)	6.8 (6.5)	6.8 (6.7)
**Refinement**			
Resolution (Å)	47.3 - 1.87	47.3 - 1.66	47.3 - 1.60
No. reflections	78587	115504	129216
R_{work} / R_{free}	12.8 / 15.9	13.8 / 16.1	14.3 / 16.7
No. atoms			
Protein	6939	6861	6819
Ligand/ion	293	327	296
Water	732	744	793
B-factors (Å^2)			
Protein	28	27	25
Ligand/ion	28	29	25
Water	39	40	38
R.m.s. deviations			
Bond lengths (Å)	0.008	0.009	0.008
Bond angles (°)	0.95	1.03	0.98

*Values in parentheses are for highest-resolution shell.

Supplementary Table 4: Plasmids used in this study.

Plasmid	Relevant characteristic(s), construction details	Reference
pBR322	Amp^R, Tet^R	(9)
pUC4K	Amp^R, Kan^R	(10)
pY179	6-kb *Eco*RI fragment of genomic DNA of *S. multivorans* subcloned into pBluescript II SK+ cut with *Eco*RI; fragment oriented inverse to *lacZ*	(11)
pTOS001	*Bam*HI/*Bgl*II-fragment of pY179 ligated with pBR322 cut with *Bam*HI; fragment oriented inverse to *tet*	This study
pTOS012	*Bam*HI-fragment (Klenow-treated) of pUC4K ligated with pTOS001 cut with *Bst*XI (Klenow-treated); fragment oriented inverse to *pceA*	This study
pTOS024	pY179 cut with *Xho*I/*Bgl*II, treated with Klenow-fragment and re-ligated	This study
pTOS036	inverse PCR using primers T68/T69 and pTOS024 as template; cut with *Nhe*I and re-ligated	This study
pTOS071	pTOS036 cut with *Nhe*I/*Afl*II and ligated with an similarly cut PCR-fragment, which was generated using primers T110/AN38 and template pY179	This study
pTOS077	*Pml*I/*Afl*II-fragment of pTOS071 ligated with pTOS012 cut with *Pml*I/*Afl*II	This study

REFERENCES

(1) Hagen, W.R. *Biomolecular EPR spectroscopy* (CRC Press / Taylor & Francis Group, Boca Raton, Fl, USA, 2009).

(2) Sjuts, H., Fisher, K., Dunstan, M.S., Rigby, S.E., Leys, D. Heterologous expression, purification and cofactor reconstitution of the reductive dehalogenase PceA from *Dehalobacter restrictus*. *Protein Expr. Purif.* **85,** 224-229 (2012).

(3) Keller, S., *et al.* Exogenous 5,6-dimethylbenzimidazole caused production of a non-functional tetrachloroethene reductive dehalogenase in *Sulfurospirillum multivorans*. *Environ. Microbiol.* **16,** 3361-3369 (2014).

(4) Neumann, A., Wohlfarth, G., Diekert, G. Purification and characterization of tetrachloroethene reductive dehalogenase from *Dehalospirillum multivorans*. *J. Biol. Chem.* **271,** 16515-16519 (1996).

(5) Fish, W.W. Rapid colorimetric micromethod for the quantitation of complexed iron in biological samples. *Methods Enzymol.* **158,** 357-364 (1988).

(6) Kräutler, B., *et al.* The cofactor of tetrachloroethene reductive dehalogenase of *Dehalospirillum multivorans* is norpseudo-B_{12}, a new type of a natural corrinoid. *Helv. Chim. Acta.* **86,** 3698-3716 (2003).

(7) Schumacher, W., Holliger, C., Zehnder, A.J.B., Hagen, W.R. Redox chemistry of cobalamin and iron-sulfur cofactors in the tetrachloroethene reductase of *Dehalobacter restrictus*. *FEBS Letters* **409,** 421-425 (1997).

(8) van de Pas, B.A., *et al.* Purification and molecular characterization of ortho-chlorophenol reductive dehalogenase, a key enzyme of halorespiration in *Desulfitobacterium dehalogenans*. *J. Biol. Chem.* **274,** 20287-20292 (1999).

(9) Bolivar, F., *et al.* Construction and characterization of new cloning vehicles. II. A multipurpose cloning system. *Gene* **2,** 95-113 (1977).

(10) Vieira, J. and Messing, J. The pUC plasmids, an M13mp7-derived system for insertion mutagenesis and sequencing with synthetic universal primers. *Gene* **19,** 259-268 (1982).

(11) Neumann, A., Wohlfarth, G., Diekert, G. Tetrachloroethene dehalogenase from *Dehalospirillum multivorans*: Cloning, sequencing of the encoding genes, and expression of the *pceA* gene in *Escherichia coli*. *J. Bacteriol.* **180,** 4140-4145 (1998).

(12) Goris, T., *et al.* Insights into organohalide respiration and the versatile catabolism of *Sulfurospirillum multivorans* gained from comparative genomics and physiological studies. *Environ. Microbiol.* **16,** 3562-3580 (2014).
